# Nonlinear dose-response relationships between psychological and behavioral factors and training quality among firefighters: a restricted cubic spline analysis

**DOI:** 10.3389/fpubh.2026.1866147

**Published:** 2026-07-31

**Authors:** Guoqing Zhu, Zongyu Liu, Huiyu Wang, Wenjia Chen

**Affiliations:** 1 School of Safety Engineering, China University of Mining and Technology, Xuzhou, China; 2 Department of Sports Science, College of Education, Zhejiang University, Hangzhou, China; 3 School of Physical Education, China University of Mining and Technology, Xuzhou, China

**Keywords:** dose-response relationship, firefighters, nonlinear, occupational health, psychological and behavioral factors, restricted cubic spline, training quality

## Abstract

**Background:**

Firefighters are exposed to multiple occupational health risks, including physical fatigue, psychological stress, and traumatic events. Although several psychological and behavioral factors have been identified as potential determinants of training quality, most prior studies assumed linear associations, which may not fully capture the true functional relationships. This study aimed to examine whether nonlinear dose-response relationships exist between six key psychological and behavioral factors and daily training quality among firefighters, and to identify inflection points and threshold effects.

**Methods:**

A cross-sectional survey was conducted among 1,587 professional firefighters from seven provinces, municipalities, and autonomous regions in China. Restricted cubic spline (RCS) models with four knots were applied to assess both overall and nonlinear associations between each psychological-behavioral variable and training quality, while controlling for the remaining variables and relevant demographic covariates (age, educational attainment, household registration type, and marital status).

**Results:**

All six psychological and behavioral variables demonstrated significant nonlinear associations with training quality: sleepiness (*F* = 15.31, *p* < 0.001), occupational stress (*F* = 11.20, *p* < 0.001), self-efficacy (*F* = 14.42, *p* < 0.001), emotional exhaustion (*F* = 11.38, *p* < 0.001), social support (*F* = 15.83, *p* < 0.001), and work-family conflict (*F* = 6.29, *p* = 0.002). The complete multivariable model explained 48.6% of the variance in training quality (R^2^ = 0.486). Dose-response curves revealed distinct threshold effects and curvature patterns for each variable.

**Conclusion:**

The associations between most psychological and behavioral factors and firefighter training quality are not simply linear; rather, they exhibit varied inflection and threshold effects. These findings have important theoretical and practical implications for the design of precision psychological intervention strategies.

## 1 Introduction

Firefighting is widely recognized as a high-risk, high-stress occupation. In the course of responding to fires, rescue operations, and emergency incidents, firefighters are chronically exposed to multiple risk factors, including physical fatigue, psychological stress, and traumatic events (1). Previous research has demonstrated that the incidence of cardiovascular disease, post-traumatic stress disorder (PTSD), and depression among firefighters is substantially higher than in the general population (2, 3). In the face of these occupational health threats, daily training not only constitutes the core mechanism through which firefighters maintain and enhance their physical fitness and technical-tactical proficiency, but also serves as a critical safeguard against casualties attributable to poor physical conditioning or operational errors (4). Accordingly, a deeper understanding of the psychological and behavioral factors affecting firefighter training quality, and the mechanisms through which they operate, is of considerable importance for optimizing training management and safeguarding operational safety.

Among the many factors influencing training quality, occupational stress has long been a prominent focus in firefighter mental health research (5). The Job Demands–Resources (JD-R) model posits that when individuals face excessive job demands without adequate buffering resources, continuous energy depletion ultimately precipitates burnout and functional decline (6). For firefighters, sources of occupational stress are diverse, encompassing task overload, role conflict, interpersonal tension, and concerns about personal safety (7). Elevated occupational stress not only directly impairs firefighters' cognitive functioning and physical performance, but also indirectly reduces their work engagement and training motivation by triggering burnout symptoms such as emotional exhaustion and depersonalization (8). Occupational burnout is another mental health concern that has received considerable attention in the firefighter literature (8–10). According to Maslach's burnout theory, occupational burnout comprises three dimensions: emotional exhaustion, depersonalization, and reduced personal accomplishment (11). Emotional exhaustion, as the core manifestation of burnout, reflects a severe depletion of emotional resources in response to sustained high-pressure environments (12). Recent studies on firefighter populations have indicated that this group faces a generally elevated risk of occupational burnout, and that distinct dimensions of burnout differentially affect firefighters‘ work capacity and health status (9, 10). A study involving Polish firefighters found that both emotional exhaustion and work disengagement significantly predicted declines in work ability, with depressive symptoms serving as a partial mediator (12).

Sleep quality is another critical determinant of firefighter training performance. Due to 24 h shift rotations and frequent overnight emergency calls, firefighters are chronically subject to sleep deprivation and circadian disruption (13). Excessive daytime sleepiness not only directly impairs attention, reaction time, and motor coordination, but is also closely associated with elevated injury risk (14, 15). A survey of 112 firefighters conducted by Carey et al. found that 59% experienced sleep deprivation and that sleep problems were significantly associated with mental health outcomes (16). Self-efficacy is a central construct in Bandura's social cognitive theory, referring to an individual's confidence in their ability to successfully execute a specific task (17). In firefighter populations, self-efficacy has been identified as an important psychological resource that buffers against occupational stress and prevents burnout (18). Firefighters with high self-efficacy tend to set more ambitious training goals, demonstrate greater persistence when confronted with difficulties, and employ more effective coping strategies during training (19). However, the precise dose-response pattern through which self-efficacy influences training quality remains unclear. Social support, as an important external resource, plays a significant protective role in firefighters' mental health and occupational functioning. Support from colleagues, family members, and supervisors can effectively buffer the adverse effects of occupational stress on mental health (20). Research has shown that firefighters with lower levels of social support are more susceptible to depressive symptoms and occupational burnout (21), and that social support serves as an important moderator of the relationship between stress and suicidal ideation (22). Work-family conflict is particularly salient among firefighters. Due to the irregularity of shift schedules and the unpredictable nature of emergency tasks, firefighters frequently struggle to balance occupational and familial responsibilities (23), and work-family conflict tends to undermine emotional wellbeing and sleep quality (13).

Importantly, the vast majority of prior studies examining associations between these psychological factors and firefighter occupational functioning have employed conventional linear regression or related linear-model techniques (10, 19, 21, 22, 24), which implicitly assumes a simple linear relationship between predictor and outcome variables. However, in practice, the influence of psychological and behavioral variables on training quality may not follow a linear trajectory; rather, it may exhibit nonlinear features such as inflection points, plateau effects, or accelerated effects (25). For instance, moderate levels of occupational stress may exert a certain activating effect on training, yet once stress exceeds a critical threshold, its adverse effects may escalate sharply—consistent with the classic Yerkes-Dodson law describing an inverted-U-shaped relationship between arousal and performance (26). Similarly, the protective effect of social support on training quality may exhibit diminishing marginal returns beyond a certain level. Neglecting these nonlinear characteristics not only risks underestimating or overestimating effect sizes, but may also mislead the development of intervention programs. Restricted cubic spline (RCS) modeling is a flexible nonlinear analytic tool that has been increasingly adopted in public health and epidemiological research (25). By placing several knots across the data range and fitting piecewise cubic polynomial functions, RCS can flexibly model the shape of the association between a continuous predictor and an outcome, while imposing linearity constraints in the tails to prevent overfitting (27). Compared with conventional approaches that categorize continuous variables for comparison, RCS preserves the full information of each variable, more accurately characterizes the curvature of dose-response relationships, and uses a nonlinearity test to determine whether the linearity assumption holds (28). RCS has been successfully applied to nonlinear dose-response studies in areas such as physical activity and mental health (29) and BMI and hypertension (30), yet its application in research on firefighter training quality remains absent.

In summary, the present study used a four-knot RCS model to systematically examine the nonlinear dose-response relationships between six psychological and behavioral variables (daytime sleepiness, occupational stress, self-efficacy, emotional exhaustion, perceived social support, and work-family conflict) and daily training quality in a sample of Chinese professional firefighters. The specific objectives were: (1) to determine whether each psychological and behavioral factor exhibits a significant nonlinear association with training quality; (2) to characterize the shape of each dose-response curve and identify potential inflection points and threshold effects; and (3) to provide evidence-based support for the development of precision and differentiated psychological-behavioral intervention strategies for firefighters.

## 2 Materials and methods

### 2.1 Study design and participants

This study employed a cross-sectional survey design. Between January and March 2026, an online questionnaire survey was administered to professional firefighters at multiple fire stations across seven provinces, municipalities, and autonomous regions in China, using convenience sampling via an online survey platform. The survey was administered through Wenjuanxing (Questionnaire Star), a widely used online survey platform in China. The electronic questionnaire link was distributed to eligible firefighters by a designated liaison officer at each participating fire station, together with standardized written instructions explaining the purpose of the study and the voluntary and anonymous nature of participation. Firefighters completed the questionnaire individually on their own smartphones during off-duty rest periods at the station; completion was self-paced, no specific time of day was imposed, and responses were not monitored by supervisors or by the research team. Inclusion criteria were: (1) formally employed firefighters on active duty; (2) aged 18 years or older; (3) regularly participating in physical fitness and skills training; and (4) providing informed consent and voluntarily participating. Exclusion criteria were: (1) individuals on extended medical leave who were unable to participate in regular training due to injury or illness; and (2) interns or temporary personnel. A total of 1,650 questionnaires were distributed. Following data cleaning, which excluded invalid responses due to anomalously short completion times, patterned responding, or missing critical information, 1,587 valid responses were retained, yielding an effective response rate of 96.2%. This study was approved by the Ethics Committee of the First People's Hospital of Xuzhou (Approval No. 2025-KY-064). All participants provided written informed consent prior to the commencement of the survey. In addition to the standardized scales described below, the questionnaire collected demographic information, including age, educational attainment, household registration type (hukou), and marital status.

### 2.2 Measures

#### 2.2.1 Daily training quality and injury prevention questionnaire

Training quality was assessed using a self-developed questionnaire entitled the *Firefighter Daily Training Quality and Injury Prevention Capacity Questionnaire*. Development of the questionnaire was grounded in a review of existing literature on firefighter training effectiveness, occupational fatigue and sleep, and safety behavior and injury prevention (4, 13, 15, 24), in-depth interviews with frontline firefighters and officers, and the requirements of the firefighter training curriculum. Following expert consultation and pilot testing, the finalized questionnaire comprises 24 items across four dimensions: (1) personal status and health habits (4 items; e.g., “Do you participate in training when fatigued or physically exhausted?”); (2) training management and facility conditions (5 items; e.g., “Do you consider the daily training load to be reasonably arranged?”); (3) training process and physical feedback (8 items; e.g., “Are you fully engaged during training?”); and (4) injury prevention knowledge and rehabilitation (7 items; e.g., “Are you aware of body parts prone to injury during training?”). All items are rated on a 5-point Likert scale, with higher total scores indicating better training quality and injury prevention capacity. Exploratory factor analysis (EFA) yielded a Kaiser-Meyer-Olkin (KMO) value of 0.933, and Bartlett's test of sphericity was statistically significant (*p* < 0.001), indicating adequate construct validity. The overall Cronbach's α for the questionnaire in the present study was 0.874, indicating good internal consistency. The full list of items is provided in Supplementary Appendix A.

#### 2.2.2 Epworth sleepiness scale (ESS)

Daytime sleepiness was assessed using the Epworth Sleepiness Scale (31). The scale comprises 8 items describing common daily situations (e.g., “sitting and reading,” “watching television”), each rated on a 4-point scale from 0 (never) to 3 (high chance of dozing), yielding a total score ranging from 0 to 24. Higher scores indicate greater daytime sleepiness. An ESS score ≥10 is conventionally regarded as the threshold for excessive daytime sleepiness, and ≥15 indicates severe sleepiness. Cronbach's α in the present study was 0.893.

#### 2.2.3 Self-efficacy scale

Self-efficacy was assessed using an adapted 9-item version of the General Self-Efficacy Scale (e.g., “I believe I am capable of coping with sudden difficulties”) (32). Items are rated on a 4-point scale from 1 (“no confidence”) to 4 (“complete confidence”), with the mean item score used as the self-efficacy score; higher scores indicate higher levels of self-efficacy. Cronbach's α in the present study was 0.972.

#### 2.2.4 Maslach burnout inventory–human services survey (MBI-HSS)

Occupational burnout was assessed using the 22-item MBI-HSS, which includes three subscales: emotional exhaustion (9 items), depersonalization (5 items), and reduced personal accomplishment (8 items) (33). All items are rated on a 7-point Likert scale. Higher scores on the emotional exhaustion and depersonalization subscales indicate greater burnout severity. Items on the reduced personal accomplishment subscale (e.g., “I can effectively deal with the problems of my colleagues”) are reverse-scored, such that higher post-reversal scores reflect lower feelings of personal achievement. Cronbach's α for the full scale in the present study was 0.891.

#### 2.2.5 Multidimensional scale of perceived social support (MSPSS)

Perceived social support was measured using the MSPSS (34). The scale comprises 12 items rated on a 5-point scale and assesses three sub-dimensions: support from significant others (including supervisors, relatives, and colleagues), family support, and friend support. Total scores range from 12 to 60, with higher scores indicating greater perceived social support. Cronbach's α in the present study was 0.977.

#### 2.2.6 Work-family conflict scale (WFC Scale)

Work-family conflict was assessed using the 18-item Work-Family Conflict Scale (35), which covers two dimensions: work interference with family (WIF; 9 items) and family interference with work (FIW; 9 items). The WIF dimension primarily assesses the negative spillover of work time, stress, and fatigue onto family life; the FIW dimension assesses the extent to which family responsibilities, emotional demands, and obligations interfere with concentration and efficiency at work. All items are rated on a 5-point Likert scale, yielding total scores ranging from 18 to 90. Higher scores indicate more severe bidirectional work-family conflict. Cronbach's α in the present study was 0.981.

#### 2.2.7 Perceived occupational stress scale

Occupational stress was assessed using a 12-item work-adapted version of the Perceived Stress Scale (36). This scale comprehensively covers three dimensions: task and sense-of-control stress, role and development stress, and interpersonal and emotional exhaustion stress, addressing aspects such as task overload, coping with emergencies, work-life imbalance, and career advancement anxiety. Items are rated on a 5-point scale, yielding total scores from 12 to 60; higher scores reflect greater perceived occupational stress. Cronbach's α in the present study was 0.985.

### 2.3 Statistical analysis

All statistical analyses were performed in R (version 4.4.1). Descriptive statistics (mean, standard deviation, median, and interquartile range) were computed for all variables. Spearman's rank correlation analysis was used to examine bivariate associations among variables. The primary analyses employed restricted cubic spline (RCS) models. In each RCS model, total training quality score was used as the continuous outcome variable; the target psychological-behavioral variable was entered in RCS form with four knots, while the remaining five psychological-behavioral variables were entered as linear terms to serve as covariates. All RCS models were additionally adjusted for four demographic covariates: age (entered as a continuous variable) and educational attainment, household registration type, and marital status (entered as categorical variables). The four knots were placed at the 5th, 35th, 65th, and 95th percentiles of the target variable's distribution. For each target variable, the following statistics were reported: (1) overall association test—evaluating whether a statistically significant association (encompassing both linear and nonlinear components) existed between the target variable and training quality (3 degrees of freedom for four knots); (2) nonlinearity test—evaluating whether a significant nonlinear component remained after removing the linear component (2 degrees of freedom); a *P*-nonlinear < 0.05 was taken as evidence to reject the linearity assumption; (3) R^2^ for the complete multivariable model, reflecting the total proportion of variance in training quality explained by all predictors; and (4) dose-response curve plots, visually displaying the predicted training quality score and 95% confidence interval across the range of each psychological-behavioral variable, facilitating identification of inflection points and threshold effects. All tests were two-sided, with a significance level of α = 0.05. RCS analyses were primarily implemented using the rms package in R.

## 3 Results

### 3.1 Sample characteristics and descriptive statistics

Four demographic variables were assessed: age, educational attainment, household registration type (hukou), and marital status. The demographic characteristics of the final sample of 1,587 firefighters are summarized in Table 1. Participant demographics were as follows: age ranged from 18 to 59 years, with a mean age of 27.24 years (SD = 6.60); regarding educational attainment, 16.6% had high school education or below, 24.6% had completed secondary vocational school, 58.6% had a college or bachelor's degree, and 0.2% held a master's degree or above; 80.7% held a rural household registration (*hukou*) and 19.3% an urban registration; 69.1% were unmarried, 29.8% were married, and 1.1% were divorced or otherwise. Descriptive statistics for all psychological-behavioral variables and training quality are presented in Table 2. The mean total training quality score was 89.58 (SD = 13.47), with a median of 90. The mean sleepiness score was 9.37 (SD = 6.29), the mean occupational stress score was 29.76 (SD = 13.21), the mean self-efficacy score was 3.28 (SD = 0.69), the mean emotional exhaustion score was 24.51 (SD = 13.70), the mean social support score was 44.17 (SD = 11.61), and the mean work-family conflict score was 47.53 (SD = 19.15).

**Table 1:** Demographic characteristics of the participants (*N* = 1,587).

Demographic variables	*N* = 1,587
Age	27.24 (6.60)
Gender	
Male	1,583 (99.7%)
Female	4 (0.3%)
Education	
High school or below	263 (17%)
Secondary vocational school	390 (25%)
Associate degree	780 (49%)
Bachelor's degree	151 (9.5%)
Master's degree or above	3 (0.2%)
Household registration	
Rural	1,280 (81%)
Urban	307 (19%)
Marital status	
Unmarried	1,096 (69%)
Married	473 (30%)
Divorced	17 (1.1%)
Widowed	0 (0%)
Cohabiting	1 (< 0.1%)

Data are presented as Mean (SD) for continuous variables (Age) and *n* (%) for categorical variables (all other variables).

**Table 2:** Descriptive statistics for all study variables (*N* = 1,587).

Variable	Mean	SD	Median	Min	Max
1. Training quality (total score)	89.58	13.47	90.00	42	120
2. Sleepiness (total score)	9.37	6.29	8.00	0	24
3. Occupational stress (total score)	29.76	13.21	34.00	12	60
4. Self-efficacy (mean score)	3.28	0.69	3.22	1	4
5. Emotional exhaustion (total score)	24.51	13.70	21.00	9	63
6. Social support (total score)	44.17	11.61	45.00	12	60
7. Work-family conflict (total score)	47.53	19.15	53.00	18	90

### 3.2 Correlation analysis

**Figure 1:**
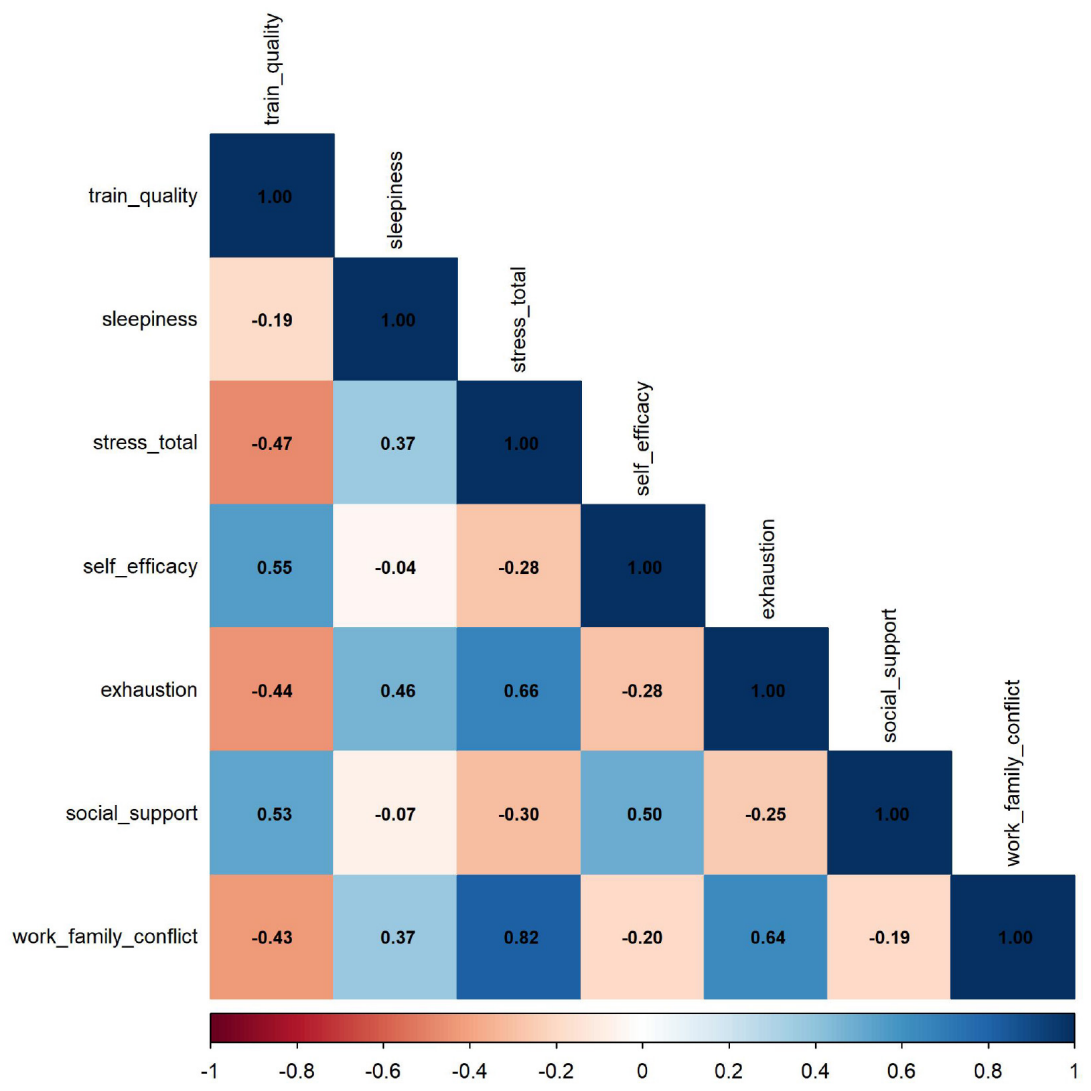
Spearman correlation heatmap among core psychological-behavioral variables and training quality among firefighters.

Spearman's rank correlation analysis results are presented in Table 3. Training quality was significantly and positively correlated with self-efficacy (*r* = 0.553, *p* < 0.001) and social support (*r* = 0.529, *p* < 0.001), and significantly and negatively correlated with occupational stress (*r* = −0.471, *p* < 0.001), work-family conflict (*r* = −0.428, *p* < 0.001), emotional exhaustion (*r* = −0.444, *p* < 0.001), and sleepiness (*r* = −0.186, *p* < 0.001). Additionally, occupational stress and emotional exhaustion showed a notably high positive correlation (*r* = 0.663, *p* < 0.001), and the correlation between occupational stress and work-family conflict was also substantial (*r* = 0.819, *p* < 0.001), suggesting potential multicollinearity among these variables. The analytic strategy of entering each target variable in spline form while controlling for the remaining variables as linear covariates in the RCS models mitigates, to some extent, the influence of multicollinearity on the results (Figure 1).

**Table 3:** Spearman correlation matrix among study variables (*N* = 1,587).

Variable	1	2	3	4	5	6	7
1. Training quality	1.000						
2. Sleepiness	−0.186	1.000					
3. Occupational stress	−0.471	0.368	1.000				
4. Self-efficacy	0.553	−0.040	−0.275	1.000			
5. Emotional exhaustion	−0.444	0.464	0.663	−0.282	1.000		
6. Social support	0.529	−0.073	−0.303	0.497	−0.251	1.000	
7. Work-family conflict	−0.428	0.375	0.819	−0.198	0.638	−0.191	1.000

Except for the correlation between sleepiness and self-efficacy (*r* = −0.040, *p* > 0.05) and the very weak correlation between sleepiness and social support (*r* = −0.073, *p* < 0.05), all remaining correlation coefficients were significant at *p* < 0.001.

### 3.3 Nonlinearity test results from RCS models

The results of the RCS nonlinearity tests for each psychological-behavioral variable are summarized in Table 4. In each RCS model, training quality was the dependent variable, the target variable was entered in spline form, and the remaining five variables were entered as linear covariates, alongside demographic covariates (age, educational attainment, household registration type, and marital status).

**Table 4:** Summary of RCS nonlinearity test results for each psychological-behavioral variable (*N* = 1,587).

Variable	Knots	Overall *F*	Overall *p*	Nonlinear *F*	Nonlinear *p*
Sleepiness	4	10.233	< 0.001	15.306	< 0.001
Occupational stress	4	9.194	< 0.001	11.199	< 0.001
Self-efficacy	4	100.558	< 0.001	14.417	< 0.001
Emotional exhaustion	4	16.166	< 0.001	11.383	< 0.001
Social support	4	75.550	< 0.001	15.833	< 0.001
Work-family conflict	4	13.112	< 0.001	6.294	0.0019

**Figure 2:**
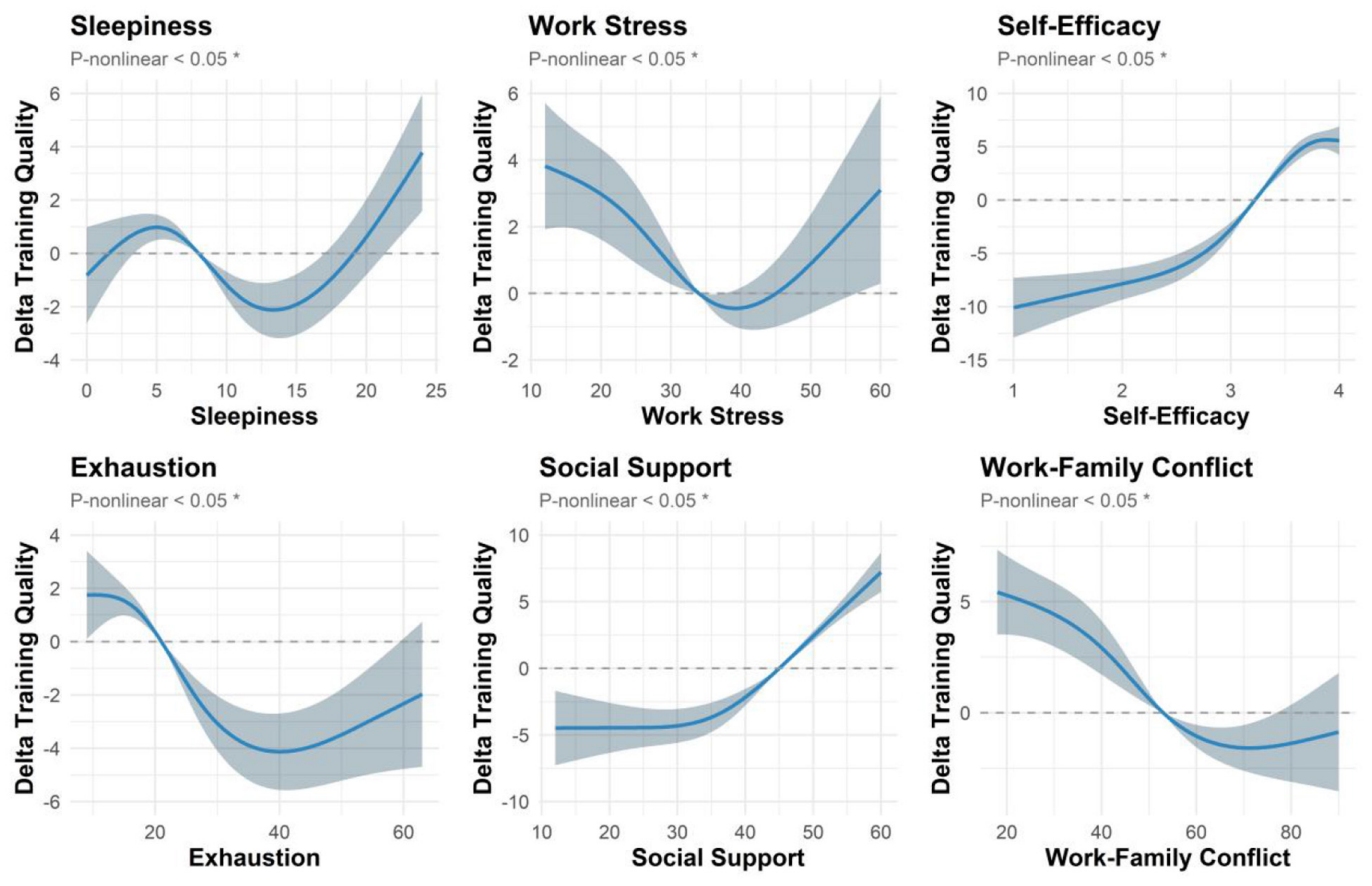
Nonlinear dose-response curves for the associations between psychological-behavioral factors and daily training quality among firefighters.

The specific findings for each variable are as follows: **(1) Sleepiness**. A significant overall association (*F* = 10.233, *p* < 0.001) and a highly significant nonlinear effect (*F* = 15.306, *p* < 0.001) were observed between sleepiness and training quality. Dose-response and piecewise regression analyses revealed that when sleepiness scores were below 16.27, training quality declined as sleepiness increased (β = −0.15, *p* = 0.008); however, once sleepiness exceeded this severe threshold of 16.27, training quality paradoxically increased (β = 0.70, *p* < 0.001). **(2) Occupational stress**. A significant overall association (*F* = 9.194, *p* < 0.001) and a significant nonlinear effect (*F* = 11.199, *p* < 0.001) were found. The dose-response curve indicated that across the broad range from low to high stress (< 46.80), increasing stress significantly impaired training quality (β = −0.16, *p* < 0.001); beyond the extreme-stress inflection point of 46.80, however, the curve exhibited a U-shaped reversal (β = 0.41, *p* < 0.001). **(3) Self-efficacy**. The overall effect of self-efficacy was highly significant (*F* = 100.558, *p* < 0.001), and the nonlinear component was also significant (*F* = 14.417, *p* < 0.001). The dose-response curve displayed a typical “threshold activation” pattern: at self-efficacy scores ≤ 2.00 (low efficacy zone), self-efficacy had virtually no significant effect on training quality (*p* = 0.595); once this psychological threshold of 2.00 was exceeded, its promotional effect on training performance showed an explosively steep increase (β = 8.04, *p* < 0.001). **(4) Emotional exhaustion**. A significant overall association (*F* = 16.166, *p* < 0.001) and a significant nonlinear effect (*F* = 11.383, *p* < 0.001) were observed. The dose-response curve showed that across most of the score range (< 59.88), emotional exhaustion continuously and steadily reduced training quality (β = −0.18, *p* < 0.001); an upward tail reversal appeared only in the extreme upper tail of the distribution (> 59.88). **(5) Social support**. The overall association for social support was significant (*F* = 75.550, *p* < 0.001), as was the nonlinear component (*F* = 15.833, *p* < 0.001). The dose-response curve revealed a clear “minimum effective dose” pattern: at lower levels of support (< 35.53), social support did not meaningfully translate into improvements in training quality (*p* = 0.743); once this threshold was exceeded, social support began to significantly and robustly enhance training quality (β = 0.45, *p* < 0.001). **(6) Work-family conflict**. A significant overall association (*F* = 13.112, *p* < 0.001) and a significant nonlinear effect (*F* = 6.294, *p* = 0.0019) were observed. The dose-response curve showed that across the broad score range from 18 to 89, greater conflict was associated with poorer training quality (β = −0.15, *p* < 0.001); similar to emotional exhaustion, an upward tail reversal appeared only near the theoretical maximum score (> 89.00) (Figure 2).

### 3.4 Model fit comparison

Likelihood ratio tests (LRT) revealed that models incorporating target variables as RCS spline terms had lower AIC values than conventional linear models for all variables, with all LRTs reaching statistical significance (*p* < 0.05 for all), indicating that the RCS specification provided a significantly better statistical fit than the corresponding linear specification for each variable. The complete multivariable model incorporating all six core psychological-behavioral variables and demographic covariates collectively explained 48.6% of the variance in firefighters' daily training quality (overall R^2^ = 0.486; LR χ^2^ = 1,055.89, *p* < 0.001).

## 4 Discussion

The central finding of this study is that all six core psychological and behavioral variables included in the analysis were significantly and nonlinearly associated with firefighter training quality, even after mutual adjustment for the other five psychological-behavioral variables and for the demographic covariates (age, educational attainment, household registration type, and marital status). This finding is consistent with previous reports of complex nonlinear associations between psychological variables and health or performance outcomes (29), and indicates that conventional linear regression is insufficient to capture the true pattern of influence these factors exert on training quality. In the public health domain, flexible modeling approaches such as RCS are widely recommended for revealing the true functional shape of the association between continuous exposures and outcomes (25). The present study innovatively applied this methodology to firefighter training quality research, filling a methodological gap in this field.

### 4.1 Nonlinear association between sleepiness and training quality

The highly significant nonlinear effect of sleepiness on training quality (*F*-nonlinear = 15.306) is consistent with the well-documented prevalence and severity of sleep problems among firefighters. Prior research has confirmed clear adverse effects of sleep deprivation on firefighters' cognitive functioning, reaction time, and motor coordination (14, 15), and excessive daytime sleepiness is closely associated with increased occupational injury risk (37). Nonetheless, the RCS curve and threshold analysis in the present study revealed an apparently counterintuitive “U-shaped reversal” phenomenon: once sleepiness exceeded the severe threshold of 16.27, it was positively rather than negatively associated with training quality. This phenomenon can be interpreted through the lens of “compensatory hyperactivation” in psychology and exercise science. According to Hockey's compensatory control model, when individuals experience extreme fatigue, they may activate higher-order executive control to mobilize “compensatory effort,” thereby maintaining or even transiently elevating performance on target tasks (38). Profoundly fatigued firefighters, driven by an instinctive fear of safety incidents on the fire ground, may forcibly focus their attention during training and rely on adrenal activation to sustain or exceed outward performance standards. This finding should by no means be interpreted as suggesting that “more fatigue is better”; rather, it reveals a dangerous borderline state in which high-risk occupational personnel are operating at the extreme limits of physical and psychological depletion.

### 4.2 Nonlinear association between occupational stress and training quality

The significant nonlinear effect of occupational stress (*F* = 11.199, *p* < 0.001) suggests that the relationship between stress and training quality is not a simple negative linear decline. Specifically, when stress scores were below 46.80, the data supported a resource depletion perspective: stress continuously degraded training performance. The U-shaped reversal observed in the extreme-stress zone (> 46.80), however, is consistent with the classic fight-or-flight response described in stress theory (39). Under extreme survival or occupational stress, the physiological arousal of firefighters may be pushed to its limits. As with the compensatory mechanism observed for sleepiness, the apparent “quality improvement” beyond the inflection point is unsustainable and exacts a substantial physiological cost. This finding cautions managers against assuming either that lower stress is always better or that firefighters can tolerate unlimited stress. In the context of the present study, an “optimal range” refers to the low-to-moderate segment of the stress continuum below the identified inflection point (< 46.80 on the 12–60 scale, approximately 1.3 standard deviations above the sample mean), within which the negative effect of stress on training quality remains gradual and manageable and the hazardous compensatory-reversal zone has not been reached. In practice, managers cannot prescribe an exact stress “dose” for each individual; instead, the optimal range can be operationalized through routine monitoring and threshold-based early warning. For example, departments may administer brief standardized stress screenings (such as the perceived occupational stress scale used here) on a regular (e.g., quarterly) basis: firefighters whose scores approach the empirical threshold could be flagged for preventive measures such as workload adjustment, recovery scheduling, and stress-management training, whereas those exceeding the threshold should receive timely professional psychological intervention and, where necessary, temporary duty modification, so as to prevent stress-induced breakdown (7). It should be emphasized that the specific cut-off identified in this study is sample-dependent and should be validated in independent firefighter samples before being adopted as a formal management criterion.

### 4.3 Nonlinear association between self-efficacy and training quality

Self-efficacy demonstrated the largest overall effect size of all variables in this study (*F*-overall = 100.558), alongside a significant nonlinear pattern (*F* = 14.417, *p* < 0.001). The nonlinear analysis confirmed the psychologically classic “threshold activation effect” (40): below 2.00 points, firefighters may be in a state of “learned helplessness,” where incremental increases in self-confidence fail to produce meaningful change in training performance; but once the critical psychological threshold of 2.00 is crossed, the motivating power of self-efficacy is released in an explosive, non-incremental fashion (18). This finding has important implications for the allocation of intervention resources: resources should be prioritized toward helping the lowest-efficacy group cross the baseline threshold, as the marginal benefit of intervention in this zone is greatest. Comprehensive intervention programs incorporating mastery experiences, peer modeling, and positive coaching feedback may be particularly valuable for this subgroup.

### 4.4 Nonlinear tail effects of emotional exhaustion and work-family conflict

Emotional exhaustion, as the core dimension of occupational burnout, exhibited a significant nonlinear effect on training quality (*F* = 11.383, *p* < 0.001), and work-family conflict displayed a highly similar curvature pattern (*F* = 6.294, *p* = 0.0019). According to conservation of resources (COR) theory, the sustained depletion of emotional resources leads to functional deterioration (41). The data in the present study showed that across most of the normal score range, both negative factors exerted a consistently stable and destructive influence, consistent with prior research (24). Although both curves showed a reversal near theoretical maximum scores (> 59.88 and > 89.00, respectively), this is most likely attributable to “spline tail oscillation” arising from sparse data at extreme values. On substantive grounds, we conclude that both emotional exhaustion and work-family conflict exert a purely negative influence on firefighter training quality across the vast majority of realistic score ranges.

### 4.5 Nonlinear association between social support and training quality

Social support demonstrated one of the strongest nonlinear effects in this study (*F* = 15.833, *p* < 0.001), as well as a prominent overall effect (*F* = 75.550). The protective role of social support for mental health among firefighters and other high-stress occupational groups has been confirmed in previous research (22, 42). Notably, the present finding both resembles and meaningfully differs from the classic finding on nonlinear effects of social support in firefighter samples. Varvel et al. (43) identified threshold effects of social support among firefighters, but described them as “diminishing marginal returns,” whereby even modest peer affirmation could substantially alleviate everyday stress at very low levels of support. By contrast, the nonlinear analysis in the present study indicates a strict “minimum effective dose” for the protective effect of social support, with an inflection point at 35.53. This discrepancy may reflect the distinctive nature of resource accumulation under extreme occupational demands: for firefighters engaged in a high-risk profession, low-level or symbolic reassurance provides no substantive benefit; only when the social support network (family understanding, peer mutual support) accumulates above a moderate-to-high threshold does it become an effective buffer against occupational stress. This suggests that when fire departments establish peer support systems or team-building initiatives, they must pursue depth and substantiveness rather than settling for superficial gestures. From a practical standpoint, departments should prioritize attention and support to firefighters with low levels of social support, such as newly recruited personnel, those working far from home, or individuals lacking family support networks, and seek to augment their social resources through peer support systems, psychological counseling hotlines, and regular team-building activities.

### 4.6 Theoretical and practical implications

From a theoretical perspective, the findings of the present study transcend the linear analytical paradigm that has predominated in prior research on firefighter training quality, revealing more complex and realistic functional relationships between psychological-behavioral factors and training quality. The existence of these nonlinear relationships supports the core assumptions of Conservation of Resources theory and Job Demands–Resources model regarding “resource loss spirals” and “gain compounding”, and provides empirical testing of these theoretical assumptions in the specific context of firefighter training. From a practical perspective, the identification of nonlinear dose-response relationships offers three key implications for the development of precision intervention strategies. First, the inflection points and threshold effects of different factors provide reference criteria for triggering intervention: management departments can use the inflection points identified from RCS curves to establish early warning indicators, so that intervention procedures are initiated when a firefighter's psychological-behavioral assessment reaches a critical threshold. Second, differences in marginal effects inform the optimal allocation of intervention resources: for positive factors such as self-efficacy and social support, priority should be given to elevating low-scoring individuals; for negative factors such as sleepiness and emotional exhaustion, priority should be given to high-risk individuals. Third, for individuals who appear to show “compensatory performance” at extremes of occupational stress or sleepiness, mandatory rest and psychological intervention should be implemented immediately to prevent sudden physiological collapse.

Building on the thresholds identified in this study, the following concrete strategies are proposed: (1) For sleepiness, departments can implement fatigue-risk-management programs combining sleep-hygiene education, strategic napping arrangements around night duties, and optimization of shift rotas, together with routine ESS screening in which scores ≥ 10 trigger enhanced monitoring and scores approaching the extreme threshold identified here trigger mandatory rest and medical evaluation. (2) For occupational stress, a tiered “screen–monitor–intervene” system is recommended: regular stress screening for all personnel; resilience training and mindfulness-based stress-reduction courses for those in the moderate range; and prompt professional counseling, workload adjustment, and temporary duty modification for those beyond the threshold, supplemented by critical-incident stress management after major deployments. (3) For self-efficacy, intervention resources should be concentrated on the low-efficacy subgroup through graded mastery-experience programs (progressively challenging but achievable training milestones), peer modeling and mentoring by experienced firefighters, and systematic positive coaching feedback. (4) For emotional exhaustion, employee-assistance programs, guaranteed recovery periods after major incidents, and periodic burnout screening are advisable. (5) For social support, structured peer-support programs with trained peer supporters, family open days and family-liaison mechanisms, and 24 h psychological hotlines should be established, prioritizing new recruits and firefighters stationed far from home. (6) For work-family conflict, family-friendly scheduling practices and communication support for firefighters' families can help reduce the bidirectional spillover.

### 4.7 Limitations and future directions

Several limitations of this study should be acknowledged. First, the cross-sectional design limits causal inference; the directionality of the nonlinear associations requires verification by longitudinal research. Second, all variables were based on self-report measures, which may be subject to common method bias and social desirability bias. Third, the sample was drawn exclusively from Chinese firefighters, and cross-cultural generalizability should be assessed with caution. Fourth, although the RCS model flexibly captures nonlinear relationships, interaction effects were not further examined (e.g., the interaction between self-efficacy and stress may produce a more complex three-dimensional response surface). Fifth, training quality was assessed using a self-developed questionnaire; although it covers multiple dimensions, its psychometric properties require further validation in broader samples.

Future studies could address these limitations in the following directions: (1) employing longitudinal or experimental designs to verify the causal direction of nonlinear relationships between key factors and training quality; (2) incorporating objective performance indicators (e.g., physical fitness test scores, training injury incidence) as supplementary measures of training quality; (3) exploring nonlinear interaction effects among multiple factors, for example by constructing a two-dimensional RCS surface for self-efficacy and stress; (4) replicating this study in firefighter samples from different countries and regions to examine the cross-cultural stability of the nonlinear association patterns; and (5) designing precision intervention programs based on the threshold information identified in this study and evaluating their effectiveness through randomized controlled trials.

## 5 Conclusion

Using data from 1,587 Chinese professional firefighters, this study systematically examined the nonlinear dose-response relationships between six core psychological and behavioral variables and training quality using restricted cubic spline models. The findings demonstrate that: (1) sleepiness, occupational stress, self-efficacy, emotional exhaustion, social support, and work-family conflict were all significantly nonlinearly associated with training quality, indicating that the conventional linearity assumption is not sufficient for these factors; and (2) self-efficacy and social support exhibited pronounced “threshold activation” and “minimum effective dose” characteristics, with positive effects increasing steeply beyond their respective inflection points, while the U-shaped reversals observed at the extreme upper tails of sleepiness and occupational stress reveal a dangerous phenomenon of “compensatory hyperactivation” under extreme physical and psychological depletion. These findings provide a robust evidence base for fire departments to move beyond “one-size-fits-all” management approaches and to develop precision and differentiated psychological-behavioral intervention strategies.

## Data Availability

The raw data supporting the conclusions of this article will be made available by the authors, without undue reservation.
