# Supplementary material for: Nonlinear dose-response relationships between psychological and behavioral factors and training quality among firefighters: a restricted cubic spline analysis
Source: Front Public Health. 2026 Jul 31;14:1866147. doi: 10.3389/fpubh.2026.1866147 (PMC13473294; doi:10.3389/fpubh.2026.1866147)
Supplement: Supplementary file 1 [file Table_1.docx]

**Supplementary Appendix A**

**The Firefighter Daily Training Quality and Injury Prevention Capacity Questionnaire**

**消防员日常训练质量与损伤预防能力问卷**

**指导语：**请根据过去 1 个月的实际感受，对以下条目进行评分（1 = 极不同意，2 = 不同意，3 = 中立，4 = 同意，5 = 极同意）。

***Instructions:*** *Please rate each of the following items based on your actual experience over the past month (1 = strongly disagree; 2 = disagree; 3 = neutral; 4 = agree; 5 = strongly agree).*

| **题号**  **Item No.** | **中文原题**  **Original Chinese Item** | **英文译文**  **English Translation** |
| --- | --- | --- |
| 1 | 您在训练时能够心无旁骛、全身心地投入训练吗？ | Are you able to stay fully focused and completely engaged during training? |
| 2 | 您对训练技术掌握的程度如何？ | How well have you mastered the required training techniques? |
| 3 | 您认为训练中的某一训练内容会对您产生紧张或恐惧吗？ | Do you feel that certain training activities cause you nervousness or fear? |
| 4 | 您在进入消防员队伍以来有抽烟的习惯吗？ | Have you been in the habit of smoking since joining the fire service? |
| 5 | 您进入消防员队伍以来有饮酒的习惯吗？ | Have you been in the habit of drinking alcohol since joining the fire service? |
| 6 | 您每天的睡眠质量如何？ | How would you rate your daily sleep quality? |
| 7 | 您会在身体异常、疲劳、体力不支的情况下参与训练吗？ | Do you participate in training when you are unwell, fatigued, or physically exhausted? |
| 8 | 您所在单位训练会针对您个人情况安排训练吗？ | Does your unit tailor training arrangements to your individual circumstances? |
| 9 | 您认为训练的内容和时长安排合理吗？ | Do you think the training content and duration are reasonably arranged? |
| 10 | 您认为日常训练负荷安排合理吗？ | Do you consider the daily training load to be reasonably arranged? |
| 11 | 您在训练开始前会进行充分的热身活动吗？ | Do you perform an adequate warm-up before training? |
| 12 | 您在训练结束后会进行充分的放松活动吗？ | Do you perform adequate cool-down activities after training? |
| 13 | 您训练结束后会感到异常疲劳或不适吗？ | Do you feel unusually fatigued or unwell after training? |
| 14 | 您所在单位会依照训练大纲进行训练吗？ | Does your unit conduct training in accordance with the official training syllabus? |
| 15 | 您觉得现有的训练场地和设施适合训练吗？ | Do you think the existing training grounds and facilities are suitable for training? |
| 16 | 您用于训练的鞋子会对训练产生影响吗？ | Do the shoes you wear for training adversely affect your training? |
| 17 | 您训练时装备的负重对训练的影响大吗？ | Does the load of the equipment you carry during training have a substantial impact on your training? |
| 18 | 训练时您佩戴使用的个人防护用品对训练损伤的预防有效吗？ | Is the personal protective equipment (PPE) you wear during training effective in preventing training injuries? |
| 19 | 您认为掌握一定的训练致伤防治知识能减少训练损伤吗？ | Do you believe that acquiring knowledge of training-injury prevention and treatment can reduce training injuries? |
| 20 | 您觉得自己需要学习训练致伤防治的有关措施吗？ | Do you feel that you need to learn measures for preventing and managing training injuries? |
| 21 | 您认为通过典型案例分析、知识讲座等方式，训练致伤可以被预防吗？ | Do you believe that training injuries can be prevented through approaches such as case analyses and educational lectures? |
| 22 | 您了解训练中易发生的损伤部位吗？ | Are you aware of the body parts most prone to injury during training? |
| 23 | 您了解训练中易出现的损伤类型吗？ | Are you aware of the types of injuries that commonly occur during training? |
| 24 | 您在训练损伤发生后会进行康复措施吗？ | Do you undertake rehabilitation measures after sustaining a training injury? |

*Note.* The questionnaire was administered in Chinese; the English translations are provided for reference only. Dimension 1 (personal status and health habits): Items 4–7; Dimension 2 (training management and facility conditions): Items 8–10, 14, and 15; Dimension 3 (training process and physical feedback): Items 1–3, 11–13, 16, and 17; Dimension 4 (injury prevention knowledge and rehabilitation): Items 18–24. Items 3, 4, 5, 7, 13, 16, and 17 are negatively worded and were reverse-scored prior to analysis, so that higher total scores indicate better training quality and injury-prevention capacity.
